# Numerical and Experimental Investigation of the Opposite Influence of Dielectric Anisotropy and Substrate Bending on Planar Radiators and Sensors

**DOI:** 10.3390/s21010016

**Published:** 2020-12-22

**Authors:** Plamen I. Dankov, Praveen K. Sharma, Navneet Gupta

**Affiliations:** 1 Faculty of Physics, Sofia University “St. Kliment Ohridski”, 1164 Sofia, Bulgaria; 2 Department of Electrical and Electronics Engineering, Birla Institute of Technology and Science (BITS), Pilani 333031, India; p2016502@pilani.bits-pilani.ac.in (P.K.S.); ngupta@pilani.bits-pilani.ac.in (N.G.)

**Keywords:** anisotropy, dielectric constant, material characterization, planar resonators, substrate bending, textile fabrics, wearable radiators

## Abstract

The simultaneous influences of the substrate anisotropy and substrate bending are numerically and experimentally investigated in this paper for planar resonators on flexible textile and polymer substrates. The pure bending effect has been examined by the help of well-selected flexible isotropic substrates. The origin of the anisotropy (direction-depended dielectric constant) of the woven textile fabrics has been numerically and then experimentally verified by two authorship methods described in the paper. The effect of the anisotropy has been numerically divided from the effect of bending and for the first time it was shown that both effects have almost comparable but opposite influences on the resonance characteristics of planar resonators. After the selection of several anisotropic textile fabrics, polymers, and flexible reinforced substrates with measured anisotropy, the opposite influence of both effects, anisotropy and bending, has been experimentally demonstrated for rectangular resonators. The separated impacts of the considered effects are numerically investigated for more sophisticated resonance structures—with different types of slots, with defected grounds and in fractal resonators for the first three fractal iterations. The bending effect is stronger for the slotted structures, while the effect of anisotropy predominates in the fractal structures. Finally, useful conclusions are formulated and the needs for future research are discussed considering effects in metamaterial wearable patches and antennas.

## 1. Introduction

Recently, many artificial materials known with their traditional applications in the human life can be considered as electrodynamic media due to the propagation of waves through them. Textile fabrics are typical examples. Most of these materials and some of their flexible polymer substitutes have been transformed into a new type of electronic components—antenna/sensor substrates due to their new applications in the wearable communication systems (antennas, sensors, radio-frequency identification or RFID, millimetre-wave identification or mmID, etc.) [1,2,3,4,5]. In this role, they look like the commercial reinforced substrates with PCB (printed circuit board) applications (a comparison has been given in [6], chapter IV). From a long time, the PCB designers have required manufacturers of the traditional reinforced substrates to provide up-to-date and reliable information on their dielectric parameters. Nowadays, the situation with the antenna designers of the wearable textile devices is almost the same—they must know the right information for the actual dielectric parameters of these specific materials as substrates. Our observations show that a lot of research papers appeared in the last several years concerning the characterization of the dielectric parameters (dielectric constant *ε_r_* and dielectric loss tangent tan *δ_ε_*) of the most popular textile fabrics [7,8,9,10,11,12]. The used methods are quite different—resonance and non-resonance—and most of them are implemented in the traditional ISM bands (typically around 2.45 GHz). However, the textile substrates differ from the reinforced substrates consist of natural and/or synthetic fibres (threads, yarns, filaments, etc.) in air and form fibrous structures with a considerably bigger variety of different cross-section views [13,14] in comparison with the simple woven or non-woven reinforced substrates. Thus, depending on the used fibre materials, their density, applied fabrication technology, and selected stitch, they act as porous materials with relatively low permittivity (*ε_r_* ~1.2–2.0), which is quite comfortable for antenna applications (the minimal dielectric constant for the reinforced substrate is typically *ε_r_* ~3.0). The other differences are that the textile fabrics are more flexible and compressible materials, the thickness and density of which can be easily changed by low mechanical pressure. One property seems common—the existence of an intrinsic planar anisotropy due to the predominant orientation of the fibres. However, the anisotropy the woven/knitted fabrics is mainly related to their mechanical properties (e.g., tensile coefficients) [1,13,14,15] and very rarely to their dielectric parameters.

Like typical artificial materials, the textile fabrics can be considered as mixtures between two or more dielectrics (reinforced fibre nets with an appropriate filling/air). In such cases, effective-media models have been developed [16], which can predict numerically the resultant isotropic dielectric constant and the dielectric loss tangent of these materials. However, the variety of technologies used for the manufacturing of these fibrous materials and the complex cross sections [5] can provoke a measurable dielectric uni- or biaxial anisotropy—direction-dependent dielectric parameters (*ε_xx_* ≠ *ε_yy_* ≠ *ε_zz_*; tan *δ_ε_xx_* ≠ tan *δ_ε_yy_* ≠ tan *δ_ε_zz_*). Our previous research [17] showed that most of the textile fabrics have typical uniaxial anisotropy: different dielectric parameters in parallel and perpendicular directions regarding to the sample surface (*ε_par_* = *ε_xx_* or *ε_yy_* ≠ *ε_perp_* = *ε_zz_*; and tan *δ_ε_par_* ≠ tan *δ_ε_perp_*), which is also typical for the wide-spread microwave reinforced substrates [6,18]. Actually, the anisotropy of both types of woven materials is an undesired property, but it should be taken into account in the RF design of different microwave (incl. antenna/sensor) components especially in the mm-wavelength range. Exactly here is the difference. Nowadays, the major manufacturers of reinforced substrate started to share information about the possible anisotropy of some of their commercial products, while the designers of wearable antennas usually completely ignore this property for the textile substrate. We found only a few papers, which comment on the dielectric anisotropy of textile fabrics. The authors of the review paper [1] considered this problem for the textile fabrics without presenting concrete data. A recent paper ([19], Table 6) investigated the influence of the percentage of the normal and in-plane (parallel) components (fibres) in the woven fabrics (at microstructural level) on the resultant dielectric constant, but without to give separate values of *ε_par_* and *ε_perp_*.

At the same time, we found another interesting fact—many papers, devoted to the dielectric characterization of textile materials, give different results for similar materials. A typical example is the measured dielectric constant of denim textile substrates, *ε_r_Denim_*. Our survey shows that the used values vary from 1.4 to 2.0 (~35% scatter) [4]. One of the reasons is the possible different types of applied weaving stitch in different cases. However, we additionally encountered a relationship between the measured dielectric constant *ε_r_Denim_* of denim fabrics on the applied measurement method. Researchers, who derive the dielectric constant from the resonance parameters of standards rectangular flat patches give values *ε_r_Denim_* ~1.59–1.67 [12,20,21,22]. In this case, the extracted dielectric constant should be close to the perpendicular one, *ε_perp_Denim_*. When the applied method is the popular coaxial dielectric probe (DAK, Dielectric Assessment Kit), the obtained parameters are typically *ε_r_Denim_* ~1.78–1.8 and beyond [23,24]. The free-space method confirms these values 1.75–2 in the frequency range 14–40 GHz [25]. Both considered methods give values close to the parallel one, *ε_par_Denim_*. Finally, the extracted dielectric constants from the microstrip ring resonator or other planar methods are typically *ε_r_Denim_* ~1.69–1.73 [23,24]. In this case, the planar methods extract the equivalent dielectric constant (see the concept developed in [26]). The equivalent dielectric constant *ε_eq_* appears for characterization of the whole substrate when the real anisotropic structure has been replaced with an isotropic equivalent. That’s why, we observe the inequality *ε_par_Denim_* > *ε_eq_Denim_* > *ε_perp_Denim_*, which is a typical situation for the woven materials, e.g., for the reinforced substrates [18,27]. Very interesting are the obtained results in [23]; they confirm the assumption above because the measured values for the equivalent dielectric constants *ε_eq_* for three textile fabrics by a ring-resonator method always are smaller than the corresponding values measured by the DAK method (*ε_par_*). Therefore, we can conclude that the anisotropy of the textile fabrics is a natural property, and its existence can explain the behaviour of their permittivity. Our investigations show that the anisotropy of the materials in the antenna project directly influences mainly the matching conditions of the patches and radome transparency [28], while then at the working frequency it indirectly slightly changes the gain, radiation patterns, efficiency and even polarization thought the anisotropic radome. The most common circumstance in the research papers considering wearable radiating components is the observation of small, moderate, and sometimes big differences between the simulated and measured resonance characteristics, explained by the authors with different experimental and simulation conditions. Our opinion is that in the most cases this effect depends on the selected by the authors values of the dielectric constant—close to *ε_perp_* (small changes for the patches resonances are observed), *ε_eq_* (suitable for the microstrip feeding lines, transformers, steps, filters, etc.) or *ε_par_* (applicable for the coplanar and slotted wearable structures) [26]. If the actual anisotropy of the used substrates is smaller than 2–3% (see below for this parameter), its influence is usually negligible.

The other important issue for the wearable antennas is the bending effect (for conformal patches) [29]. A part of the research papers dealing with the wearable antennas on textile fabrics and polymers usually include additional information for the effect of bending at typical radii, compliant with the human body [30,31,32,33]. A measure for the degree of bending is the curvature radius *R_b_* (*R_b_* is the radius of an imaginary cylinder to which the antenna is bent) or bending angle *θ_b_* = *L* (or *W*)/*R_b_*, where *L* and *W* are the length and width of the rectangular patch antenna [34]. Most of the papers simply registered the bending effect on the working frequency and/or frequency bandwidth (usually a decrease of the resonant frequency) and rarely on the gain and radiation pattern. Sometimes, unexpected discrepancies are detected between the simulated and measured results from the bending [32] explained by imperfect measurements. Only a few researchers provide discussions for the nature of the bending effect. When the measurements are well performed, the obtained results are useful for understanding the bending effect. For example, the results obtained in the paper [30] give the information that the thickness of the flexible substrate is important for the degree of the bending influence. For the substrate as a flexible felt (*ε_r_* = 1.3) with thickness *h_S_* = 0.5–12 mm, the optimal thickness for minimizing the effect of bending over the frequency shift is about 6 mm. Very helpful results for the bending effect on rectangular patch antenna on denim substrate are presented in [35]. The parameters of this material with thickness *h_S_* = 2 mm are chosen to be *ε_r_Denim_* = 1.6 and tan *δ_ε_Denim_* = 0.01 at 2.4 GHz. For the first time, the authors definitely show by simulations that the resonance frequency of the lowest-order TM_10_ mode in the rectangular patch antenna should continuously increase with increasing of the bending radius *R_b_*—with a relatively low degree for the width-bent patches and with a higher degree—for the length-bent patches. However, the measurement results slightly differ from the simulations, as relatively big ripples appear in the experimental frequency shifts: ±2.5 MHz for width-bent and ±85 MHz for length-bent patches (compared to the resonance frequency ~2.4 GHz for the flat patches). Nevertheless, the tendency for increasing of the resonance frequency is visible. The authors commented that this behaviour was not expected from simulations. They attribute this discrepancy to other physical properties that the conductive textile was subjected to upon bending that were not correctly replicated in simulations.

In our paper [36], we supposed for the first time that both effects (bending of the flexible substrate and its anisotropy) can simultaneously affect the resonance behaviour of the resonance patches. There we presented some preliminary experimental results for a rectangular resonator with isotropic substrates, but the influence of the substrate anisotropy was not separately investigated. We cannot find other research papers, where the anisotropy and bending are considered in parallel, excepting some calculations of the input impedance [37] and additionally return losses S_11_ and mutual coupling [38] in cylindrically conformal patch antennas on anisotropic substrates.

In this paper, we continue to investigate more deeply the opposite impacts of the dielectric anisotropy and bending of the substrate on the resonance characteristics of planar radiators. We follow the same strategy in this paper—not to consider fed patches and antennas, but to examine pure resonant structures and to avoid any parasitic influence of the feeding lines. This paper includes new experimental and simulation results for the frequency shift of the modes in planar rectangular resonators and their modifications, which makes possible the separation between the effects of anisotropy and bending and the independent characterization of the degree of these effects. In the Materials and Methods section, two experimental and numerical methods have been used for determination of the uniaxial anisotropy of the textile fabrics. A methodology for accurate measurements of the bending effects on the resonance characteristics of the planar resonator has been described. Then, an efficient procedure is introduced for creating suitable 3D models of planar resonators for separate numerical investigations of the bending and anisotropy and both effects together. Data for the measured anisotropy of several selected for the research flexible anisotropic and isotropic materials are presented. In the Results and Discussions section, very interesting results are obtained and discussed for the separate and simultaneous influence of the anisotropy and bending for materials with different anisotropy and for conformal resonance structures bent at different radii. The results for the influence of the anisotropy and bending on several planar resonators with sophisticated shapes are added—for slotted rectangular patches, fractal structures, and resonators with defected grounds. Finally, the origins of the considered competitive effects on the resonance planar structures are discussed and explained and useful conclusions are offered. A possible future work has been formulated.

## 2. Numerical and Experimental Methods and Materials

The aim of this research is to investigate numerically and experimentally the possible competitive influences of the uniaxial anisotropy and bending of textile substrates on the resonance performances of wearable planar radiators. Therefore, in this section, we describe all applied experimental and numerical methods for the determination of the substrate anisotropy and reliable characterization of the bending effect in these radiating structures. The selected materials and their important characteristics for the research have been obtained.

### 2.1. Two-Resonator Method for Measurement of the Uniaxial Anisotropy of Textile Fabrics

**Figure 1:**
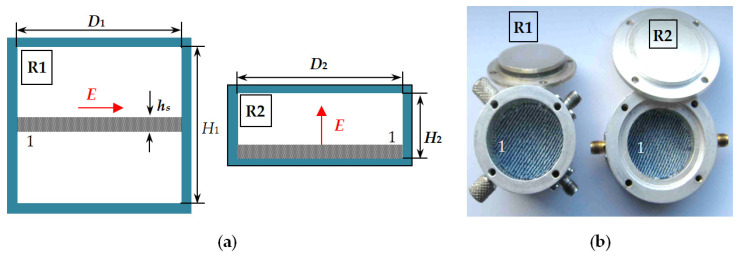
Two-resonator method: (**a**) Pair of resonators for measurement of parallel (R1) and perpendicular (R2) dielectric parameters of disk samples in cylindrical TE (R1) and TM-mode (R2) resonators; (**b**) Photography of resonators R1 and R2 with denim textile sample 1; E—electric field.

The considered below method has been proposed in [27] and applied for anisotropy characterization of a variety of materials [19]. In this paper, it has been applied for the determination of the pairs of parameters, *ε_par_*; tan *δ_ε_par_* and *ε_perp_*; tan *δ_ε_perp_*, of textile fabrics. Figure 1a schematically presents the idea of the used method: a textile disk sample is placed sequentially in two resonators, which are designed to support either symmetrical TE_0*mn*_ modes (*m* = 1, 2, 3, …; *n* = 1, 2, 3, …) in the cylinder marked as R1 or symmetrical TM_0*m*0_ modes (*m* = 1, 2, 3, …) in the cylinder marked as R2 with mutually perpendicular E fields—parallel to the sample surface in R1 or perpendicular to this surface in R2. The sample is placed in the middle of R1 and on the bottom of R2 ensuring the best conditions for the excited TE or TM modes to be influenced by the sample and these modes to be maximally separated (e.g., the resonators heights to be *H*_1_ ~ *D*_1_ and *H*_2_ < *D*_2_ and the coupling probes to be orientated to excite only TE modes in R1 or TM modes in R2). The sample diameter *d_S_* is chosen to coincide with the resonator diameters *d_S_* ~ *D*_1,2_. In this case, the extraction of the dielectric parameters can be accurately performed by the analytical model described in [27,39]. In short, the measurement procedure is as follows. First, the resonance characteristics are measured (resonance frequency *f*_0_ and unloaded quality factor *Q*_0_) of each TE or TM mode under interest in the empty R1 or R2 resonator. This step makes possible a fine determining the equivalent resonator diameters *D*_1,2*eq*_ and equivalent wall conductivity *σ*_1,2*eq*_ of both resonators, which considerably increases the accuracy of the next measurements. The second step includes measurements of the resonance characteristics (*f_ε_* and *Q_ε_*) of the same TE or TM modes (well-identified) in the R1 or R2 resonators with a sample. Finally, the set of obtained data ensures the determination of the parallel dielectric constant *ε_par_* and dielectric loss tangent tan *δ_εpar_* in resonator R1 and determination of the perpendicular dielectric constant *ε_perp_* and dielectric loss tangent tan *δ_εperp_* in resonator R2. The measurement uncertainty has been evaluated as relatively small [27]: 1–1.5% for *ε_par_*, 3–5% for *ε_perp_*, 5–7% for tan *δ_εpar_* and 10–15% for tan *δ_εperp_* in the case of 0.5–1.5 mm thick substrates with dielectric constants ~1.3–5 in the Ku band. The main source of the pointed inaccuracy is the uncertainty for the determination of the sample thickness. Another circumstance is the selectivity of the considered method; due to the E-fields orientation the cylinder resonators measure the corresponding “pure” parameters (parallel ones in R1 and perpendicular ones in R2) with selectivity uncertainty less than ±0.3–0.4% for the dielectric constant and less than ±0.5–1.0% for the dielectric loss tangent in a wide range of substrate anisotropy and thickness [39].

### 2.2. Numerical Models for Determination of the Dielectric Constant and Anisotropy of Textile Fabrics as Dielectric Mixtures

#### 2.2.1. Limits for the Dielectric Parameters of Mixed Textile Threads

There exists a big variety of technologies to mix or blend two or more types of textile threads from different materials and with different mechanical properties, which makes possible to obtain new fabrics with desired specific elasticity moduli and stiffness and to control the stability of these properties. Due to these purposes, textile engineers have developed different models and effective- medium theories for reliable characterization of these structures [13,14,15,16,40,41,42]. Our survey shows that these models with some modifications could be successfully applied also to the electromagnetic properties of the textile fabrics dielectric mixtures, as it has been done in [16].

**Figure 2:**
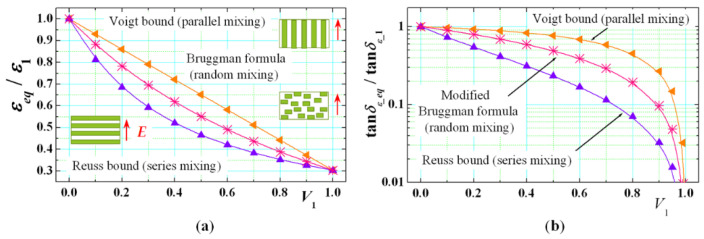
Minimal and maximal bounds for the normalized resultant equivalent dielectric constant *ε_eq_*/*ε*_1_ (**a**) and normalized equivalent dielectric loss tangent tan *δ_ε_eq_*/tan *δ_ε_*__1_ (**b**) of two mixed dielectrics with isotropic parameters *ε*_1_/tan *δ_ε_*_1_ and *ε*_2_/tan *δ_ε_*_2_ and normalized volumes *V*_1_ and *V*_2_ (*V*_1_ + *V*_2_ = 1) (insets: of parallel, series and random mixing regarding the direction of the applied E field).

The simplest models (as the first stage of approximation, if the details of geometry are ignored) give the so-called upper and lower bounds of the resultant dielectric constant and loss tangent on the base of the modified Reuss (iso-strain) and Voigt (iso-stress) bound models (for series or parallel layered mixtures). The Bruggman formula [14] presents relatively accurate approximation for near-to-isotropic materials:

(1) $$\varepsilon_{eq}=\frac{\left(\varepsilon_{1}+u)(\varepsilon_{2}+u\right)}{V_{1}(\varepsilon_{2}+u)+V_{2}(\varepsilon_{1}+u)}-u;\;0\leq u\leq \infty ,\;V_{1}+V_{2}=1$$

where *ε_eq_* is the scalar isotropic equivalent dielectric constant of the mixture, *ε*_1_ and *ε*_2_ are the dielectric constants of the mixed threats, *V*_1_ and *V*_2_ are the corresponding normalized volumes, and *u* ⊂ (0; ∞) is a parameter which depends on the method of mixing. Three cases could be derived from this expression depending on the type of mixing: for series mixing *u* = 0 (Reuss bound); for parallel mixing *u* = ∞ (Voigt bound) and for random mixing, *u* = (*ε*_1_*ε*_2_)^1/2^ (Bruggman curve). All these curves for the normalized dielectric constant are plotted in Figure 2a.

The Equation (1) is a complex one; it can be rewritten also for a direct calculation of the corresponding dielectric loss tangents bounds (modified Bruggman formula); see the dependencies in Figure 2b:

(2) $$\tan\delta_{\varepsilon \_eq}=\frac{\left(\tan\delta_{\varepsilon }{}_{1}+v)(\tan\delta_{\varepsilon }{}_{2}+v\right)}{V_{1}(\tan\delta_{\varepsilon }{}_{2}+v)+V_{2}(\tan\delta_{\varepsilon }{}_{1}+v)}-v;\;0\leq v\leq \infty ,$$

where tan *δ_ε_eq_* is the isotropic equivalent dielectric loss tangent of the resultant fabrics, tan *δ_ε_*_1_ and tan *δ_ε_*_2_ are the dielectric loss tangent of the mixed/blended threats, and *v* ⊂ (0; ∞) is a new parameters; now we have again *v* = 0 for series mixing, *v* = ∞ for parallel mixing, however, *v* = [*ε*_1_*ε*_2_ (tan *δ_ε_*_1_ + tan *δ_ε_*_2_)]^−1/2^ for random mixing. We have selected a concrete synthetic material for the presented examples in Figure 2a,b—Polyester threads (*ε*_1_ ≅ 3.4; tan *δ_ε_*_1_ ≅ 0.005) mixed with air (*ε*_2_ = 1.0; tan *δ_ε_*_2_ = 0). However, the predicted anisotropy by Equations (1) and (2) is too large, does not take into account the concrete sizes and shapes of the threads and therefore, the results do not correspond to the realistic textile fabric. The survey of other effective-media analytical expressions for the resultant permittivity in different mixtures, presented in [43], show that they also cannot give the actual anisotropy.

Therefore, in this paper, we accepted another more realistic approach. Most of the textile fabrics can be considered as complex fabrics of cylindrical single or multi-fibre threads (a short survey on the Internet of the free microscopic images of the popular fabrics illustrates well the predominant existence of the cylindrical cross-section shape of the threads). Such an approach is very popular in the mechanical models of the textile fabrics [13,14,15], but also applicable for characterization and modelling of their dielectric properties [10,19,44]. In this research, a similar approach has been accepted. In the next subsection, we present an effective numerical model for accurate prediction of the real anisotropy of textile fabrics on the base of cylindrical unit cells.

#### 2.2.2. Numerical Models for Evaluation of the Dielectric Anisotropy of the Textile Fabrics

**Figure 3:**
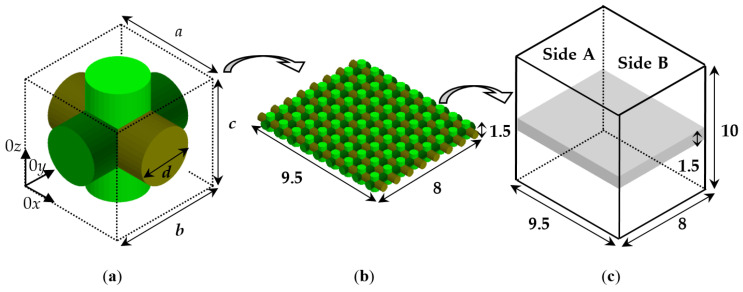
(**a**) Unit cell *a* × *b* × *c* with cylindrical threads of diameter *d*; (**b**) constructed artificial sample with repeated unit cells in a hosting isotropic substrate (air); (**c**) equivalent sample in a rectangular box, which is a quarter part of the whole resonator with symmetrical boundary conditions on Side A and B.

**Figure 4:**
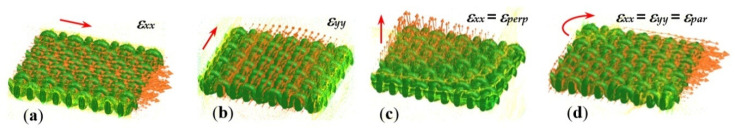
Artificial sample (3D-woven fabrics) in a rectangular resonator (not shown), which supports different modes with mutually perpendicular E fields (red arrows) along the axes: 0*x*, 0*y*, 0*z* and in the plane 0*xy* in the Ku-band (modes: TE_011_ (**a**); TE_101_ (**b**); TM_010_ (**c**); TE_111_ (**d**).

The degree of anisotropy can be predicted for artificial textile fabrics by the numerical method introduced in [17]. The idea of this method is to build a unit cell by two or more isotropic cylindrical fibres (threads), to reproduce it in a hosting isotropic substrate (e.g., air) and to put the whole sample in a rectangular resonator, which supports TE and TM modes with exited E fields in three mutually perpendicular directions. The simulations are performed by electromagnetic simulators (HFSS^®^ in this case). Figure 3 illustrates the selected unit cells with three mutually perpendicular cylinders of equal diameter *d*. The concrete unit cell is a prism with sides *a* = *b* = 1.0; *c* = 1.5 mm. They form a rectangular sample with dimensions 9.5 × 8 × 1.5 mm and this sample is placed in the middle of a rectangular box with dimensions 9.5 × 8 × 10 mm. Actually, this box is one-quarter part of a rectangular resonator with dimensions 19 × 16 × 10 mm, which support TE and TM mode in the Ku and K bands depending on the diameters, filling and dielectric constant of the threads. The resonator with a sample is solved in “eigenmode” option of the used HFSS simulator (calculating the resonance frequency *f_r_* and the unloaded quality factor *Q*), where appropriate symmetrical boundary conditions are accepted at side A and B of the box: “symmetrical E-field” for TE modes and “symmetrical H-field” for TM modes. Thus, the considered resonator with 1.5-mm thick artificial textile sample supports the following mode of interest, illustrated in Figure 4 for a 3D-woven textile sample: (a) TE_011_ mode with resonance frequencies in the interval 19.3–21 GHz (E field along 0*x*); (b) TE_101_ mode; 21.9–23.5 GHz (E field along 0*y*); (c) TM_010_ mode; 11.6–12.2 GHz (E field along 0*z*); (d) TE_111_ mode; 17.6–18.7 GHz (E field in plane 0*xy*). The considered set of modes makes it possible the extraction of the dielectric constants and dielectric loss tangent of the investigated textile samples in different directions, considered as samples with bi- or uniaxial symmetry as it is shown in Figure 4. The concrete resonator dimensions are chosen relatively small (to facilitate simulations). However, larger dimensions can be selected for lower-frequency ISM bands. The only rule is the size of the unit cell to be smaller than the free-space wavelength to ensure homogenization of the artificial structure at a given frequency. As quantitative measures are used, the parameters Δ*A_ε_*, Δ*A*_tan_*_δε_* for the degree of the dielectric anisotropy of the resulting (equivalent) dielectric constant/loss tangent for bi-/uni-axial anisotropy are calculated by the following expressions:

(3) $$\Delta A_{\varepsilon \_xx,yy}=2(\varepsilon_{xx,yy}-\varepsilon_{zz})/(\varepsilon_{xx,yy}+\varepsilon_{zz}),$$

(4) $$\Delta A_{\tan\delta \varepsilon \_xx,yy}=2(\tan\delta_{\varepsilon \_xx,yy}-\tan\delta_{\varepsilon \_zz})/(\tan\delta_{\varepsilon \_xx,yy}+\tan\delta_{\varepsilon \_zz}).$$

Independent extraction of the resultant dielectric constant along all three axes is possible after the replacing of the anisotropic sample under test with an equivalent isotropic sample (as in Figure 3c). The procedure is as follows. After the selection of each unit cell and the construction of the whole artificial 3D sample (see below), placed in the middle of the selected resonator, the resonance frequency *f_r_* and Q factor of the corresponding mode can be obtained by simulations in “eigenmode option”. Then, the anisotropic structure is replaced with an equivalent prism of the same dimensions and by tuning the corresponding isotropic values *ε_eq_* and tan *δ_εeq_*, a coincidence should be reached (typically <1%) between both simulated pairs *f_r_* and Q for the anisotropic sample and its isotropic equivalent. Thus, the corresponding dielectric parameters of the biaxial anisotropic textile samples can be obtained by using the excited modes in Figure 4a–c, while the parameters for the uniaxial anisotropic textile samples (most of the cases) can be obtained by using the modes in Figure 4c,d.

**Figure 5:**
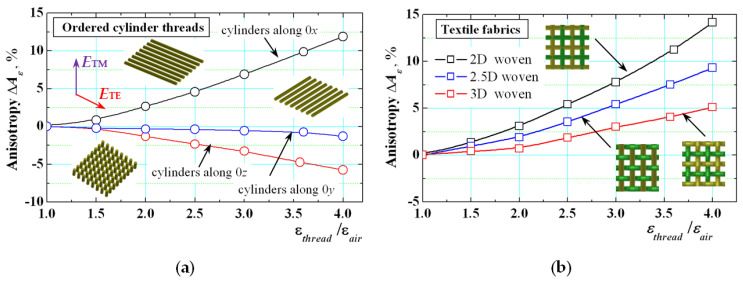
Dielectric constant anisotropy of artificial textile samples versus the ratio between the dielectric constant of the threads and air *ε_thread_*/*ε_air_*: (**a**) for ordered straight cylinders, orientated along axes 0*x*, 0*y* or 0*z*; (**b**) for 2D, 2½D and 3D woven fabrics in Ku-band (see Figure 6) (Arrows: E fields of the used TE and TM modes).

The described procedure is effective and enough accurate for preliminary prediction of the anisotropy of different artificial materials. In the paper [17], some preliminary results have been obtained for several artificial woven and knitted tactile fabrics, but the method has been successfully applied for many other materials (incl. 3D printed) [6]. In this paper, we present new results to show how the anisotropy depends on the structure and threads’ orientation of the textile fabrics and to establish the origin of this property. Some attempts to find such relations have been done in [19] but without to present quantitative results. In the beginning, three simple structures have been constructed as in paper [17] based on ordered single cylinders built from the unit cell in Figure 3 of diameter 0.5 mm and distance between their axes 1.0 mm. The cylinders are consistently orientated along 0*x*, 0*y* and 0*z* axes. Applying the modes from Figure 4a,c with electric fields orientated along 0*x* or 0*z* axis, the described model makes it possible to determine the dependence of the dielectric constant anisotropy Δ*A_ε_xx_* versus the ratio *ε_thread_*/*ε_air_* presented in Figure 5 (a). The parameter Δ*A_ε_xx_* increases with the ratio *ε_thread_*/*ε_air_* increasing, but in different ways. When the cylinders are orientated along 0*x* (as the electric field *E*_TE_ of the exited mode), Δ*A_ε_xx_* has large positive values (~8% for *ε_thread_* = 3.4). Contrariwise, when the cylinders are orientated along 0*z* (*E*_TM_), Δ*A_ε_xx_* has negative values (~−4.5%). These values strictly correspond to the relative volume portions of the treads orientated along 0*x* (*E*_TE_) and 0*z* (*E*_TM_) in these simple cases (detailed geometrical calculations are not performed at this stage of the research). Only when the cylinders are orientated along 0*y* (perpendicularly to *E*_TE_ and *E*_TM_, the parameter Δ*A_ε_xx_* is close to 0 (i.e., the sample behaves as almost isotropic one).

**Figure 6:**
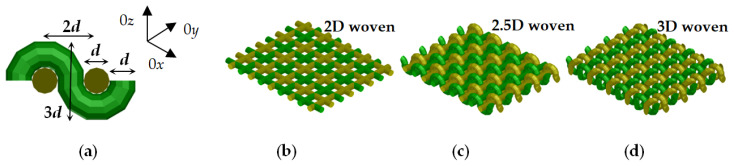
(**a**) Woven threads of corresponding dimensions; three types of woven fabrics: (**b**) 2D woven; straight threads along 0*x*, 0*y*; (**c**) 2.5D woven; straight threads along 0*y*, wavy threads along 0*x*; (**d**) 3D woven; wavy threads along 0*x*, 0*y* (all threads are with equal dielectric constant *ε_thread_* = 3.4).

Very interesting are the results for the uni-axial anisotropy (obtained by the modes from Figure 4c,d) of constructed three artificial woven fabrics (shown in Figure 6). They are conditionally named 2D, 2.5D and 3D woven samples due to the applied straight and/or wavy threads (see the figure captions of Figure 6). The behaviour of the uni-axial parameter Δ*A_ε_* of the 2D-woven sample is close to this one of the pure cylinders along 0*x*—Figure 5b. However, when the portion of the threads with orientation along 0*z* axis increases (for 2.5D and especially for 3D-woven samples) applying wavy threads, the anisotropy becomes smaller, from 10% (for 2D woven samples) to 7% (2.5D) and 3.5% (3D) for *ε_thread_* = 3.4. This result shows that the dense woven fabrics have relatively small anisotropy, close to the realistically measured values of 4–6% for most of the textile fabrics. However, their anisotropy exists and can be taken into account in the design of different wearable devices, when the final design accuracy is important and for the higher 5G frequency bands.

### 2.3. Procedure for Accurate Measurements of Bent Planar Resonators on Textile Fabrics

**Figure 7:**
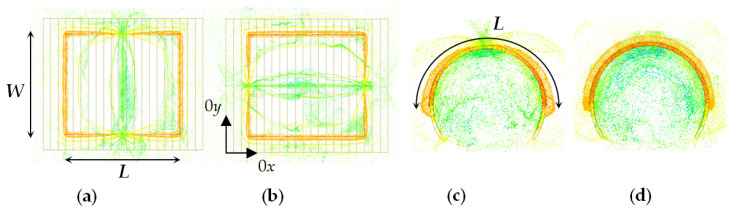
Simulated E-field pattern in microstrip resonators: (**a**,**b**) TM_10_ and TM_01_ in a flat resonator; (**c**,**d**) TM_10_ and TM_01_ in a bent resonator. Legend: *L*—length; *W*—width.

**Figure 8:**
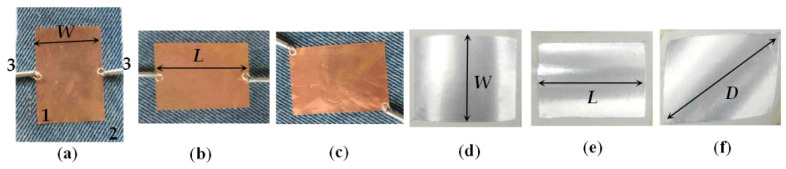
(**a**–**c**) Pair of magnetic coaxial loops placed on the length, width, and diagonal of the planar resonator; (**d**–**f**) length (L)-bent, width (W)-bent and diagonal (D)-bent microstrip resonators. Legend: 1—resonator; 2—substrate; 3—pair of magnetic coaxial probes.

The accurate measurement of bent wearable structures is not an easy task. There appear strong mechanical changes during the bending—deformations in the substrates; deformations in the metal layout (it should always tightly cover the substrate); the feeding lines can affect the resonance behaviour. Following the strategy in this paper to investigate only pure resonance structures, we apply coaxial probes to excite the lowest-order resonances in the planar structures. Figure 7 represents the simulated E-field pattern of the first two planar modes in flat and bent microstrip resonators. Coaxial probes from electric type (short coaxial pin orientated along the E field) should be put close to the E-maximums. However, in this research, we apply more stable coaxial magnetic loops placed close to the H-maximums of the magnetic field of the corresponding mode (Figure 8a for TM_10_ mode; Figure 8b for TM_01_ mode and Figure 8c for both modes). The measurements are performed by a vector network analyzer in the L and S bands in transmission regime. The place and the orientation of the loops are tuned during the measurements until the transmission losses S_21_ increase more than −40 dB. At these conditions, the resonance frequency practically does not depend on the loop proximity and the measured resonance frequencies are enough accurate.

In this research, we apply self-adhesive 0.05-mm thick metal (Al or Cu) folio to form the resonator layout. We start measurements in a flat position of the resonator and then measure the bent resonator with continuously decreasing bending radius. The resonator substrates are bending over a set of smooth metallic cylinders with radii *R_b_* from 80 to 12.5 mm. Three types of bending are applied—length-(L), width-(W) and diagonal-bent (D) resonators—see the illustrations in Figure 8. When we bend, special care is taken to ensure that the metallization remains well adhered to the substrate and that it does not detach itself. Therefore, measurements are performed only for decreasing bending radius and not in reverse order. Each of the pointed types of bending is realized with a new fresh resonator folio. In this research, the results are presented for the ratio between the resonance frequencies for the bent and flat resonators.

### 2.4. Numerical Models for Investigations of Bent Planar Resonators on Anisotropic Substrates

**Figure 9:**
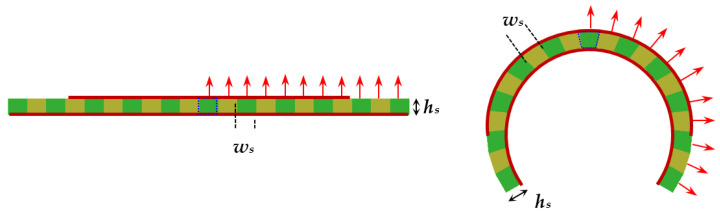
Flat and bent microstrip resonators on substrate constructed by sliced prisms, each with own anisotropic properties. Arrows represent the normal direction in each slice in flat and curved substrates.

**Figure 10:**
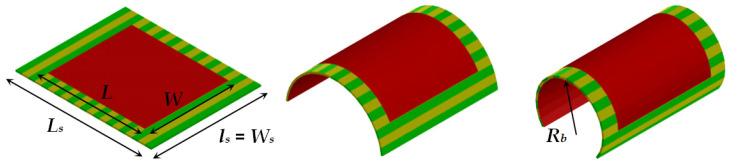
3D view of flat and bent microstrip resonators of length *L* = 30 and width *W* = 26 mm on sliced substrates with length *L_s_* = 42 and width *W_s_* = 34 mm (last two cases with bending radius *R_b_* = 14.3 and 9.6 mm).

Most of the modern electromagnetic simulators have options for the introduction of anisotropic materials. However, in the case of conformal planar structures, this is not easy to perform directly, when the substrate has been introduced as a single object and to be sure that the anisotropy is accurately described. Therefore, we chose a geometrical approach. The anisotropic substrate is divided into several equal slices with a form of prisms (with rectangular cross-section view for the flat resonators and with trapezoidal cross-section view for the flat resonators). The slices have equal anisotropic properties as the whole substrate, but the parallel and perpendicular directions used to determine the uniaxial anisotropic dielectric parameters can be controlled now for each slice with the change of the bending radius—as it is sown in Figure 9 for the half of structures. In this research, the concrete width *w_s_* of the slices is chosen to be *w_s_* = 2 mm but can be decreased for thicker substrates or smaller bending radii for better fitting of the cross-section of the bent substrate. The other sizes are height *h_s_* and length *l_s_* = *W_s_*. The 3D views of flat and bent microstrip resonators on sliced anisotropic substrates are presented in Figure 10. During the bending, we satisfy the rule to keep the resonator dimensions *L* and *W*. However, the ground and the slices may undergo some deformations.

### 2.5. Materials Used in the Research

Based on the purposes of the paper, several types of materials have been selected. One of the groups consists of several textile and polymer samples with different measured degrees of anisotropy (Δ*A_ε_* from 4.3 to 10.3) by the two-resonator method. The measured results for the pairs of parameters *ε_par_*/tan *δ_εpar_* and *ε_perp_*/tan *δ_εperp_*, as well as for the uniaxial anisotropy Δ*A_ε_*/Δ*A*_tan_*_δε_* are presented in the upper part of Table 1. The other group includes several flexible isotropic substrates, selected for measurement of the pure bending effect. The measured anisotropy of these materials is very small, Δ*A_ε_* < 1%. The last two groups have representatives of relatively flexible reinforced substrates and soft artificial ceramics. Their anisotropy Δ*A_ε_* varies in a big interval—8.2–24.5%.

## 3. Results and Discussion

Three types of results and corresponding discussions are presented in this section. First, numerical and experimental results are presented for the pure bending effect in planar resonators on flexible isotropic and near-to-isotropic substrates (Section 3.1). The next step is to verify with results the assumption that the bending effect and substrate anisotropy have opposite impacts on the wearable radiators and sensors (Section 3.2). Finally, the simultaneous bending and anisotropy influence is investigated for several sophisticated planar resonators with magnetic slots, defected grounds and for Koch fractal resonators (Section 3.3).

### 3.1. Pure Bending Effect

As we mentioned in the Introduction, the investigated bending effect in wearable planar patches and devices usually has been masked by other phenomena, not considered in the simulations [32,33]. We try to solve these problems applying experimentally-proven pure flexible isotropic substrates, using pure resonance structures (to minimize the effects of the feeding lines) and follow an accurate measurement procedure described in Section 2.3.

**Figure 11:**
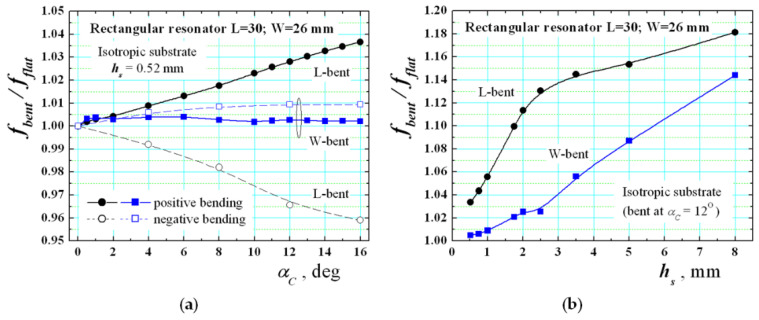
Numerical dependencies of the ratio between the resonance frequencies *f_bent_*/*f_flat_* of the lowest-order TM_10_ mode for bent and flat rectangular resonators on isotropic substrate versus (**a**) the curvature angle *α_C_* between the substrate slices and (**b**) substrate thickness *h_s_*. The isotropic dielectric constant is chosen to be 3.0, but its concrete value has negligible influence. Positive and negative curvature angles are used.

**Figure 12:**
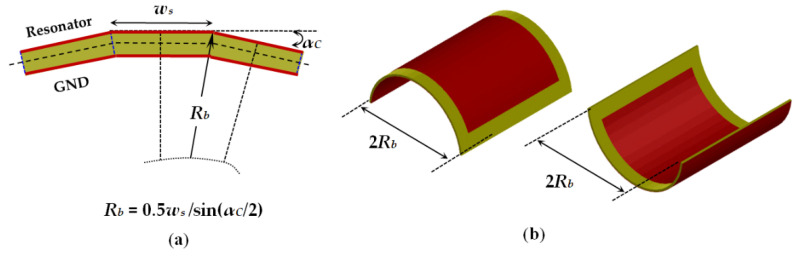
(**a**) Definition of the relation between the curvature angle *α_C_* and bending radius *R_b_*; dashed line: middle line in the resonator substrate, where the effective electrical length of the resonator is formed; (**b**) resonance structures on positive (+*α_C_*) and negative (−*α_C_*) bent substrate (the bending radius *R_b_* is always determined to the side of the resonator layout).

First, Figure 11a presents the dependencies of the ratio *f_bent_*/*f_flat_* between the resonance frequencies for the lowest-order TM_10_ mode for bent and flat rectangular resonators on pure isotropic substrate versus the curvature angle *α_C_* between the neighbour slices used to construct the substrate. This is a new measure for the bending degree, which is more comfortable in our research. Figure 12 illustrates the relationship between the bending radius *R_b_* and the introduced curvature angle *α_C_* (e.g., *α_C_* = 4° corresponds to *R_b_* = 28.7 mm; *α_C_* = 8°—*R_b_* = 14.3 mm; *α_C_* = 12°—*R_b_* = 9.6 mm, etc.).

The presented results show the expected fact (mentioned in [35]) that the resonance frequency of the L-bent resonator increases in comparison to the flat case for pure isotropic substrates. The dependence is not exactly linear. At the same time, the effect on the bending is relatively small for W-bent resonators, which is also an expected result. These dependencies correspond to the classical “positive” bending (*α_C_* > 0). What happens during the bending? The material undergoes mechanical deformations, e.g., stretching at the top (to the resonator) and shrinking at the bottom area (to the ground). In our model, we take into account this effect by changing the cross-section shape of the separate slices from rectangular to trapezoidal (illustrated in Figure 9 and Figure 12a). The narrow side of the trapezoid is orientated to the ground of the resonance structure. Thus, the model confirms the assumption that the electrical length *L_E_* of the L-bent resonator decreases in comparison to the geometrical length *L* (illustrated with the dashed line in Figure 12a). The standing wave of the lowest order TM_10_ mode is located exactly along the curvature in the L-bent structures (see Figure 7a,c) and it explains the increase of the resonance frequency when the curvature angle *α_C_* increase. Contrariwise, during the W-bending the standing wave is located in a perpendicular direction and the influence of the bending is negligible, especially for thin substrates.

Figure 11a presents also the bending effect for the “negative” bending (*α_C_* < 0). It is just the opposite and this confirms the origin of the bending effect for the wearable structures. Now, the narrow side of the trapezoid of each slice is orientated to the resonator layout of the resonance structure and in this case, the effective electrical length *L_E_* of the L-bent resonator increases in comparison to the geometrical length *L* and the corresponding resonance frequency decreases. This type of bending is rarely used and not discussed in detail.

Finally, Figure 11b additionally shows the variations of the bending effect in substrates with different thickness. Now, the effect considerable increases for a thickness interval of 0.5–2.5 mm and then saturation appears for L-bent structures (relatively strong increase is observed also for W-bent structures at bigger thicknesses). However, we cannot observe here the existence of an optimal thickness, where the bending effects are minimized as shown in [30].

**Figure 13:**
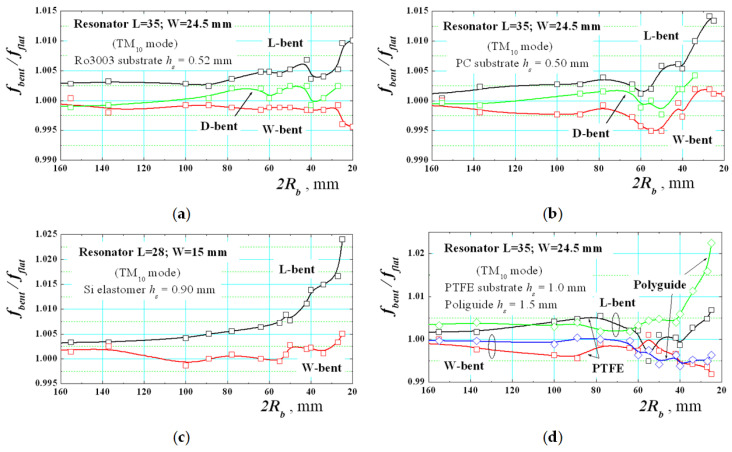
Experimental dependencies of the ratio between the resonance frequencies *f_bent_*/*f_flat_* of the lowest-order TM_10_ mode for bent and flat rectangular resonators on several isotropic substrates versus the bending radius *R_b_*: (**a**) Ro3003; (**b**) PC; (**c**) commercial silicone elastomer; (**d**) PTFE and Polyguide^®^ Polyflon (http://www.polyflon.com; dielectric parameters 2.05/0.00045).

The next step is to prove experimentally these tendencies. Figure 13 gives a set of measurement results for the ratio *f_bent_*/*f_flat_* of the lowest-order TM_10_ mode in bent and flat rectangular resonators on several isotropic substrates versus the bending radius *R_b_*. Three types of dependencies are shown—for L-, W and D-bent resonators. All the results are close to results from the numerical simulations in Figure 11a (D-bent resonators are not simulated). They depend on substrate flexibility and deformations. The best results are got for the well-flexible silicone elastomer (*h_s_* = 0.9 mm), Figure 13c. Good results are obtained by Ro3003 substrate (*h_s_* = 0.52 mm), Figure 13a; however, at small bending radii, this soft substrate undergoes technological stretching and *f_bent_* slightly decreases. The harder substrate PC (*h_s_* = 0.5 mm) shows better stability at low *R_b_*. The results for the soft PTFE substrate (*h_s_* = 1.0 mm) deviate from the theoretical dependencies due to the poor adhesion properties of this materials to the metal folio. However, the PTFE-like material with the commercial mark Polyguide^®^Polyflon (*h_s_* = 1.5 mm) demonstrates better behaviour. In all presented cases, the curves for D-bent substrates (moderate influence) lie between the curves for L-bent (upper curves; stronger influence) and W-bent substrates (lower curves; smaller influence). Thus, we can conclude that the experimental results for the pure bending effect on planar resonators on isotropic substrate fully confirm the numerical simulations, taking into account the possible substrate deformation during the bending on very small radii *R_b_*.

### 3.2. Investigation of the Simultaneous Effects of Anisotropy and Bending of Planar Resonators

**Figure 14:**
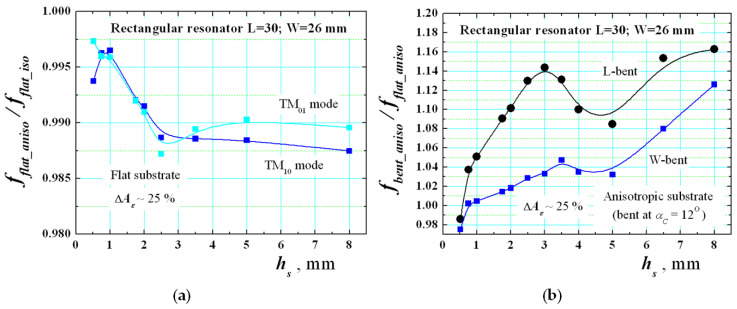
(**a**) Numerical dependencies of the ratio *f_flat_aniso_*/*f_flat_iso_* between the resonance frequencies of modes TM_10_ and TM_01_ for flat rectangular resonators on anisotropic and isotropic substrate versus the substrate thickness *h_s_*. (**b**) Numerical dependencies of the ratio *f_bent_aniso/_f_flat_aniso_* of mode TM_10_ for L-/W-bent and flat rectangular resonators on anisotropic substrates versus the substrate thickness *h_s_*.

The main expected results in the research are included in this section. In the beginning, it is important to evaluate the effect of anisotropy in flat resonators. Figure 14a shows the simulated dependencies of the ratio *f_flat_aniso_*/*f_flat_iso_* between the resonance frequencies of modes TM_10_ and TM_01_ for flat rectangular resonators on anisotropic (Δ*A_ε_* ~25%) and isotropic substrates versus the substrate thickness *h_s_*. The effect is visibly weak. Only for relatively thick substrates does the resonance frequency shift due to the anisotropy influence with 1–1.5%, which explains why this property is not so popular in the patch antenna design. The explanation is easy—the parallel E fields (to have a noticeable influence of the *ε_par_* component) appear only close to the edge of such wide planar structure and the relative effect is practically negligible in comparison to the microstrip line [26].

**Figure 15:**
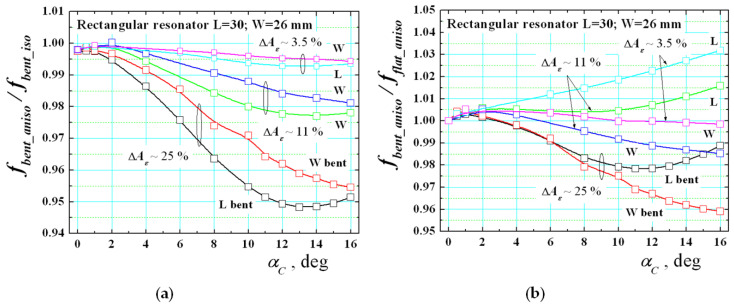
(**a**) Numerical dependencies of the ratio *f_bent_aniso_*/*f_bent_iso_* between the resonance frequencies of mode TM_10_ in L/W-bent resonators on anisotropic and isotropic substrates (*h_s_* = 0.52) versus the curvature angle *α_C_*; (**b**) Numerical dependencies of the ratio *f_bent_aniso/_f_flat_aniso_* of mode TM_10_ in L-/W-bent and flat rectangular resonators on anisotropic substrates versus the curvature angle *α_C_*.

However, we expect a stronger effect when the resonators are bent. To perform deeper research, a set of bent resonators with different curvature angle are simulated by the help of the 3D models shown in Figure 9 and Figure 10. First, the ratio *f_bent_aniso/_f_bent_iso_* is shown in Figure 15a between the resonance frequencies of mode TM_10_ for L-/W-bent rectangular resonators versus the curvature angle *α_C_*. The substrate anisotropy Δ*A_ε_* is chosen to be small (~3.5%), moderate (~11%) and big (~25%). This ratio is not measurable, but it shows in a pure form the effect of anisotropy in bent resonators. The results give the useful information, obtained for the first time, that this influence is considerably bigger in comparison with the flat case (up to −5% shifts down). One can see from the presented dependencies that the influence of the substrate anisotropy decreases the resonance frequency in comparison to the hypothetical case of an isotropic bent substrate. Therefore, we can conclude that the effect of the anisotropy of the substrate is just opposite to the effect of bending (as it is shown in Figure 11a). This was our preliminary hypothesis, and it can be considered as proven numerically. Therefore, one can expect that both effects can strongly change the behaviour of these dependencies.

Figure 15b presents the ratio *f_bent_aniso/_f_flat_aniso_* between the resonance frequencies of mode TM_10_ for L-/W-bent rectangular resonators on anisotropic substrates versus the curvature angle *α_C_*. Now, this ratio is measurable and can be verified experimentally. The new dependencies show that the resonance frequency shift in resonator on realistic (anisotropic) substrates may have as positive, as well as negative signs depending on the actual parameter Δ*A_ε_*, which is impossible for pure isotropic substrates. We also investigate the influence of the substrate thickness *h_s_* on corresponding ratio *f_bent_aniso/_f_flat_aniso_*. Figure 14b presents curves for L- and W-bent resonators at curvature angle *α_C_* = 12°. The results show that the bending effect can compensate the anisotropy influence for thicker substrates to some degree. It is interesting to note that as in [30], we observe the fact that for mediate thicknesses (named “optimal thickness” in [30]) the effect of anisotropy decreases the bending effect; this property probably depends on the curvature angle *α_C_* and not investigated in detail.

**Figure 16:**
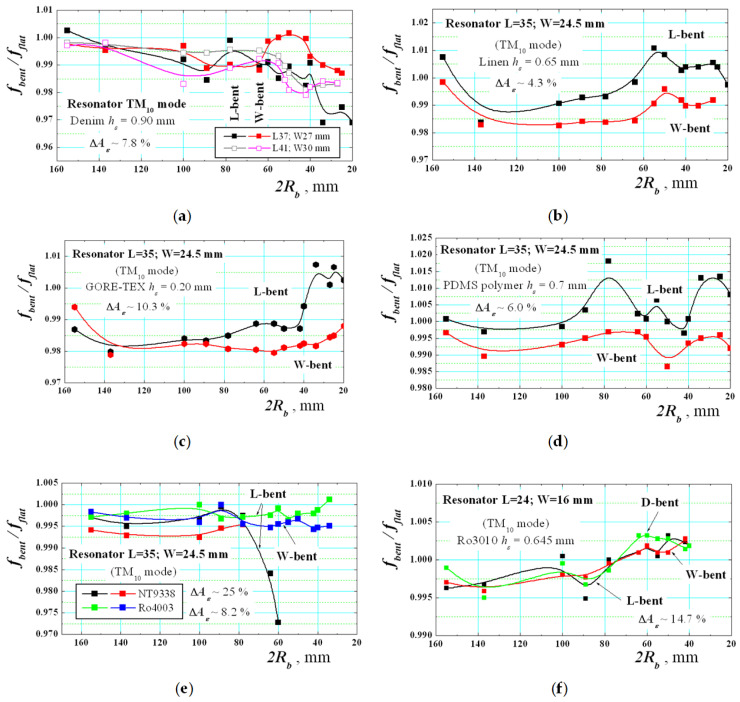
Experimental dependencies of the ratio between the resonance frequencies *f_bent_*/*f_flat_* of the lowest-order TM_10_ mode for bent and flat rectangular resonators on several anisotropic substrates versus the bending radius *R_b_*: (**a**) Denim; (**b**) Linen; (**c**) commercial textile fabrics GORE-TEX^®^; (**d**) PDMS; (**e**) NT9338, Ro4003; (**f**) Ro3010.

Let’s now present some experimental dependencies for bent resonators on anisotropic substrates, selected in Section 2.5. The measurement results for the ratio *f_bent_*/*f_flat_* of the TM_10_ mode in bent and flat rectangular resonators versus the bending radius *R_b_* are presented in Figure 16. They differ from the dependencies shown in Figure 13 for isotropic substrates. In anisotropic case, more or less expressed ripples in the resonance shifts is observed in both L- and W-bent resonators below the resonance frequencies of the corresponding flat resonators (as in paper [35]), which is practically impossible for the isotropic case when accurate measurement procedure has been applied. Therefore, all these cases confirm the simultaneous effects of the anisotropy and bending of used substrates. Very typical are the curves for the textile fabrics denim, linen and commercial multilayer GORE-TEX^®^ and for the flexible polymer PDMS with a small degree of stretching. Similar behaviour is observed for three relatively flexible commercial reinforced substrates: Ro4003; NT9338 and soft ceramic Ro3010. However, the course of dependences here is affected also by the non-plastic deformation in these substrates, which does not allow bending at very small radii. Of course, all presented experimental curves cannot be directly compared with the theoretical ones in Figure 15b due to the difficulties to satisfy the perfect measurement conditions especially at small bending radii, but the trends that reveal the impact of the anisotropy together with the bending effect in wearable structure is obvious.

### 3.3. Effects of Anisotropy and Bending on More Sophisticated Planar Resonators

The fact, that the substrate anisotropy visible influences together with the bending the resonance behaviour of such simple structure as the rectangular resonator gives us the idea to verify this influence for more complicated planar resonance structures on anisotropic substrates. In this subsection, several resonance structures with slots, defected grounds and Koch fractal contours are numerically investigated to verify the effects of anisotropy and bending in the L and S bands.

**Figure 17:**
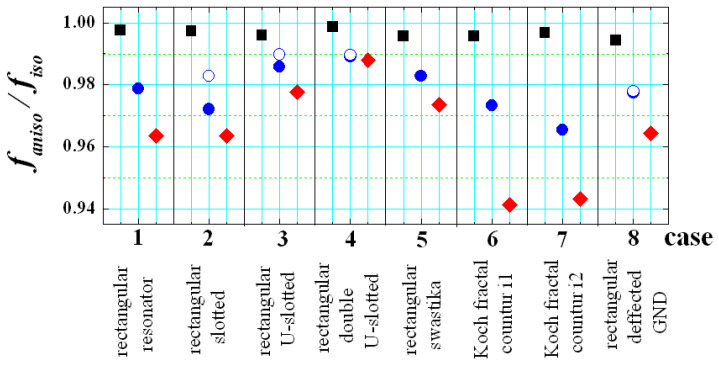
Simulated values of the ratio *f_aniso_*/*f_iso_* between the resonance frequencies of the lowest-order mode in several planar resonance structures with dimensions 30 × 30 mm on anisotropic and isotropic substrates (*h_s_* = 0.52; Δ*A_ε_* ~ 25%) (this ratio gives the pure effect of anisotropy). The shapes of the considered structures are presented in Figure 19 and Figure 20. The first column for each case corresponds to a flat structure, second—bent at *α_C_* = 8°; third—bent at *α_C_* = 12°; Solid and hollow points correspond to two mutually perpendicular orientations (V & H) of the structure during the bending (when this is possible).

**Figure 18:**
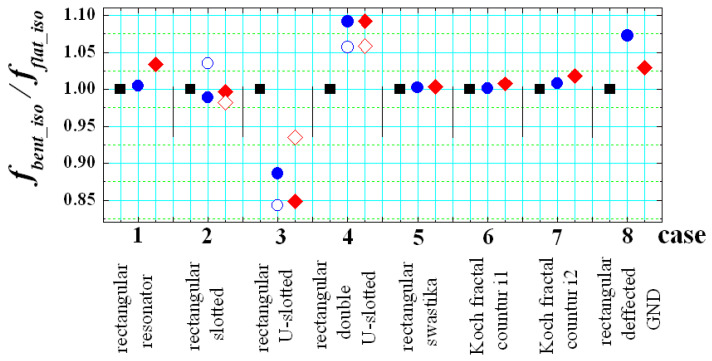
Simulated values of the ratio *f_bent_iso_*/*f_flat_iso_* between the resonance frequencies of the lowest-order mode in several planar resonance structures with dimensions 30 × 30 mm on isotropic substrates (*h_s_* = 0.52; Δ*A_ε_* ~ 25%) (this ratio gives the pure effect of bending). The shapes of the considered structures are presented in Figure 19 and Figure 20. The first column for each case corresponds to a flat structure, second—bent at *α_C_* = 8°; third—bent at *α_C_* = 12°; Solid and hollow points correspond to two mutually perpendicular orientations (V & H) of the structure during the bending (when this is possible).

Two types of results are presented in Figure 17 and Figure 18. We again investigate the ratio *f_aniso_*/*f_iso_* between the resonance frequencies of the lowest-order mode for each structure on anisotropic and isotropic substrate (Figure 17). This ratio is a measure of the pure effect of anisotropy. The second type of result is for the ratio *f_bent_iso_*/*f_flat_iso_* between the resonance frequencies of the lowest-order mode in the same planar resonance structures (bent and flat) on isotropic substrates (Figure 18). Now, this ratio is a measure of the pure effect of bending.

**Figure 19:**
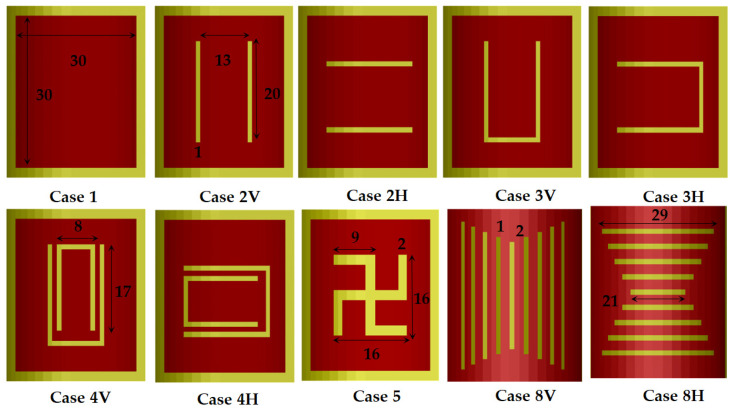
Top view of several resonance structures (bent at *α_C_* = 8°) with dimensions in mm: Case 1—resonator (30 × 30); Case 2—resonator with two slots (V—vertical orientation; H—horizontal orientation); Case 3—resonator with U-shaped slot (V&H); Case 4—resonator with double U-shaped slot (V&H); Case 5—resonator with swastika slot; Case 8—resonator with a defected ground (V&H).

In the beginning, several rectangular patches with magnetic slots have been considered. These structures are usually applied for a widening of the bandwidth of the corresponding planar patches in comparison to the standard planar patch (Case 1). They include several types of slots (see also Figure 19): Case 2—resonator with two slots [45]; Case 3—resonator with U-shaped slot [30,46]; Case 4—resonator with double U-shaped slot [47]; Case 5—resonator with swastika slot [48]. The structures are not optimized; their dimensions are presented in Figure 19 and are compliant with the used grid of the sliced substrates. The results from Figure 17 show that the effect of the anisotropy decreases (4–1%) with adding the listed slots in the resonator layout. These slots are placed relatively far from the edges, where the parallel E fields exist and the anisotropy cannot change effectively the electrical dimensions of the slotted resonators. At the same time, the pure bending effect is larger, especially for the resonators with U-shaped slots—Figure 18.

The considered defected-ground resonator [49] is also not strongly influenced by the anisotropy (2–3%); the effect is comparable with the effect in the planar resonator with a standard ground. However, the pure bending effect is strong, especially for horizontally-placed slots in the defected ground (case 8H; the increase is more than 25–50%; the values are not shown in Figure 18, because are out of the scale).

**Figure 20:**
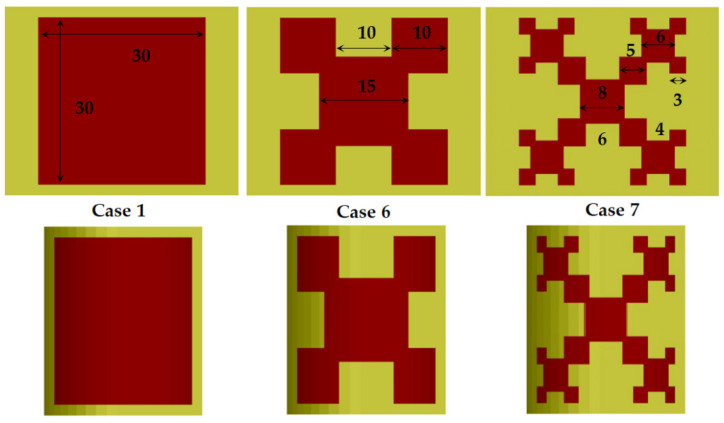
Top view of the first three iterations of Koch fractal contours as a planar resonator (flat—first row and bent at *α_C_* = 8°—second row) with dimensions in mm: Case 1—iteration 0; Case 6—iteration 1; Case 7—iteration 2.

Actually, only the considered fractal resonators demonstrate relatively big resonance shifting due to the anisotropy of the substrates—Figure 17 (4–6%), while the pure bending effect is even smaller than in the case of the standard planar resonator (iteration i0)—Figure 18. We investigate the first and second iterations (i1, i2) of classical Koch fractal contours [50], performed on flat and bent substrates—Figure 20. The reason for the increased anisotropy influence is that the portions of the parallel E fields increase considerably with the iteration number of the fractal resonators, which provokes stronger resonance frequency shift down (when *ε_par_* > *ε_perp_*). A similar effect can be expected in most of the metamaterial surfaces used in the wearable flat and bent antennas, which is the objective of our future work.

## 4. Conclusions

The main objective of this study has been accomplished—to prove the opposite influences of the effects of anisotropy and bending on the resonance characteristics of flexible wearable structures. The advantage of this paper is that both effects have been separated in the numerical simulations, which makes it possible to evaluate the degree and sign of the resonance frequency shifts of simple rectangular planar resonators on anisotropic and isotropic substrates in flat and bent states. All simulations and the obtained experimental results show that the pure bending effect, performed only by experimentally-verified isotropic substrates, increases the resonance frequency of the bent rectangular resonators in comparison to the flat ones and proves the origin of this effect in a pure form. Contrariwise, the presented numerical analysis shows that the anisotropy (the existence of direction-dependent dielectric constants *ε_par_* and *ε_perp_* of the textile materials and similar woven substrates) has just an opposite influence—the resonance frequency of the flat or bent rectangular resonators on anisotropic substrates always decreases (when *ε_par_* > *ε_perp_*) in comparison to the same structures on pure isotropic substrates. The last effect is not directly measurable, but it gives the expected pure effect of the substrate anisotropy, which depends on the degree of anisotropy Δ*A_ε_* and the actual bending radius *R_b_*. The combined effects, anisotropy and bending, lead to a more complicated behaviour of the investigated resonance structures when the bent and flat rectangular resonators are considered—as positive, as well as negative resonance frequency shifts.

Now, these combined effects are fully measurable. Applying well-selected flexible anisotropic substrates (including textile fabrics), the resonance shifts in bent and flat resonance structures are measured in the L and S bands. The obtained dependencies for bending radii *R_b_* from 80 up to 10 mm show as increasing (as for the pure bending effect), as well as decreasing of the resonance frequencies (the last phenomenon is theoretically impossible for pure bending effect). Due to the mechanical deformations in the same of the materials during the bending, the obtained dependencies do not fully coincide with the numerical ones, but the tendencies for the opposite influence of the anisotropy and bending are considered as proven. The obtained results explain well the observed dependencies by other authors, even the existence of optimal substrate thicknesses, where the effect of bending (but we add the anisotropy, too), could be minimized. Of course, the last phenomenon depends on the concrete bending radius and anisotropy degree.

Encouraged by the results obtained for such a simple structure as the planar rectangular resonator on wearable substrates, we performed a useful numerical study for the combined effects of anisotropy and bending for more sophisticated structures—planar resonators with slots and defected grounds and fractal resonators. In some of them, the bending effect predominates (resonators with slots and defected grounds), while in the other structures, e.g., the fractal resonators with increased iteration number, the effect of the anisotropy is stronger than the bending effect. These new results determine also the direction of our future research—to investigate complex metamaterial structures for wearable antennas performed on anisotropic substrates at different bending angles. The proposed experimental and numerical methods by the applied TE/TM modes for reliable determination of the direction-dependent equivalent dielectric parameters of different metasurfaces ensure the preliminary determination of the anisotropy of these structures, while the proposed methods for parallel investigation of the effects of bending and anisotropy—the accurate behaviour of metasurfaces with accurate curvature. This is a new scheme of research concerning such meta structures, which will be developed in our future work.

## Figures and Tables

**Table 1:** Measured dielectric parameters and anisotropy of selected materials for this research (averaged values for the frequency interval 6–13 GHz).

Material	*h_s_*, mm	*ε_par_*/tan *δ_ε___par_*	*ε_εperp_*/tan *δ_ε___perp_*	Δ*A_ε_*/Δ*A*_tan_*_δε_*, %
*Textile and polymer samples*				
Denim	0.90	1.74/0.048	1.61/0.030	7.8/38
Linen	0.65	1.65/0.043	1.58/0.044	4.3/−2.3
Waterproof fabric with breathability GORE-TEX^®^	0.20	1.53/0.0057	1.38/0.0043	10.3/28
Polydimethylsiloxane (PDMS)	0.70	2.73/0.022	2.57/0.019	6.00/15
*Flexible isotropic and near-to-isotropic samples*				
Polytetrafluoroethylene (PTFE)	0.45	2.05/0.00027	2.04/0.00026	0.49/3.8
Polycarbonate (PC)	0.50	2.77/0.0056	2.76/0.0055	0.36/1.8
Silicone elastomer	0.90	2.21/0.0010	2.19/0.0008	0.91/22
Ro3003	0.51	3.00/0.0012	2.97/0.0013	1.0/−8
*Relatively flexible anisotropic reinforces substrates*				
Ro4003	0.21	3.67/0.0037	3.38/0.0028	8.2/28
NT9338	0.52	4.02/0.005	3.14/0.0025	24.6/67
*Relatively flexible anisotropic soft ceramics*				
Ro3010	0.645	11.74/0.0025	10.13/0.0038	14.7/−41

## Data Availability

Data is contained within the article. More detailed data and data presented in this study are available on request from the corresponding author. Part of them could be included in the Final reports to the corresponding funding organizations.
